# Clinical value of preoperative serum CA 19-9 and CA 125 levels in predicting the resectability of hilar cholangiocarcinoma

**DOI:** 10.1186/s40064-016-2181-x

**Published:** 2016-04-30

**Authors:** Hai-Jie Hu, Hui Mao, Yong-Qiong Tan, Anuj Shrestha, Wen-Jie Ma, Qin Yang, Jun-Ke Wang, Nan-Sheng Cheng, Fu-Yu Li

**Affiliations:** Department of Biliary Surgery, West China Hospital of Sichuan University, Chengdu, 610041 Sichuan Province China; Department of Respiratory Medicine, West China Hospital of Sichuan University, Chengdu, 610041 Sichuan Province China; Department of General Surgery, Gandaki Medical College, Pokhara, Nepal

**Keywords:** Hilar cholangiocarcinoma, Carbohydrate antigen 19-9 (CA 19-9), Carbohydrate antigen 125 (CA 125), Resectability

## Abstract

**Background:**

To examine the predictive value of tumor markers for evaluating tumor resectability in patients with hilar cholangiocarcinoma and to explore the prognostic effect of various preoperative factors on resectability in patients with potentially resectable tumors. Patients with potentially resectable tumors judged by radiologic examination were included. The receiver operating characteristic (ROC) analysis was conducted to evaluate serum carbohydrate antigenic determinant 19-9 (CA 19-9), carbohydrate antigen 125 (CA 125) and carcino embryonie antigen levels on tumor resectability. Univariate and multivariate logistic regression models were also conducted to analysis the correlation of preoperative factors with resectability.

**Results:**

In patients with normal bilirubin levels, ROC curve analysis calculated the ideal CA 19-9 cut-off value of 203.96 U/ml in prediction of resectability, with a sensitivity of 83.7 %, specificity of 80 %, positive predictive value of 91.1 % and negative predictive value of 66.7 %. Meanwhile, the optimal cut-off value for CA 125 to predict resectability was 25.905 U/ml (sensitivity, 78.6 %; specificity, 67.5 %). In a multivariate logistic regression model, tumor size ≤3 cm (OR 4.149, 95 % CI 1.326–12.981, P = 0.015), preoperative CA 19-9 level ≤200 U/ml (OR 20.324, 95 % CI 6.509–63.467, P < 0.001), preoperative CA 125 levels ≤26 U/ml (OR 8.209, 95 % CI 2.624–25.677, P < 0.001) were independent determinants of resectability in patients diagnosed as hilar cholangiocarcinoma.

**Conclusions:**

Preoperative CA 19-9 and CA 125 levels predict resectability in patients with radiological resectable hilar cholangiocarcinoma. Increased preoperative CA 19-9 levels and CA 125 levels are associated with poor resectability rate.

## Background

Hilar cholangiocarcinoma is a devastating malignant neoplasm with a poor prognosis and is generally diagnosed at an advanced stage (Baton et al. 2007; Xiong et al. 2015). Curative bile duct resection combined with hepatectomy, caudate lobectomy, lymph node dissection and even vascular resection and reconstruction represents the only curable method that can prolong the survival of patients diagnosed with hilar cholangiocarcinoma (Ramacciato et al. 2010; Hidalgo et al. 2008; Neuhaus et al. 2003; Miyazawa et al. 2006). However, hilar cholangiocarcinoma has the propensity of longitudinal diffusion along the bile duct mucosa, which makes the curative surgery difficult (Cheng et al. 2012). Moreover, hilar cholangiocarcinoma is located in a peculiar anatomic position and tends to invade major adjacent structures including the hepatic parenchyma, the caudate lobe, the portal vein, the hepatic artery and the peripheral nerve system, resulting in low resectability chances, high postoperative recurrence, and poor survival outcome (Saxena et al. 2011; Li et al. 2011; Ercolani et al. 2010; Burke et al. 1998). Some tumors are considered resectable in view of preoperative imaging evaluation. However, the intraoperative findings of biliary involvement and vascular invasion often do not match with the preoperative assessment. How to improve the predictive role of tumor resectability—is still a puzzled problem faced by surgeons. Curative resectability rate is usually very dismal (<40 %) and most of the patients are conducted palliatively (Saxena et al. 2011; Launois et al. 2000; Jarnagin et al. 2001, 2005).

Previous studies indicated serum tumor markers such as CA 19-9, CA 125 and CEA are important reference factors for the diagnosis and prognosis of gastrointestinal malignant neoplasms and are conventionally detected before and after surgery (Juntermanns et al. 2010; Wang et al. 2015). These serum tumor markers are also widely used in predicting the resectability of gastrointestinal malignancies (Luo et al. 2013; Brown et al. 2015). As for hilar cholangiocarcinoma, the clinical predictive value of these parameters remains indistinct and there is an ongoing debate about the validity of these tumor markers for hilar cholangiocarcinoma. In addition, the serum CA 19-9 level depends on the Lewis phenotype and about 5–14 % of the population is Lewis negative (Le^a−b−^), which was characterized by CA 19-9 nonsecretory (Chen et al. 2015; Liu et al. 2015; Cai et al. 2014). Patients with Lewis negative (Le^a−b−^) cannot secrete the CA 19-9 even when they possess a malignant tumor, which is associated with poor survival outcome even after curative resection. However, previous studies almost ignored this significant factor while analyzing the prognostic effect of preoperative serum CA 19-9 levels on hilar cholangiocarcinoma. Furthermore, previous studies did not consider the impact of obstructive jaundice and cholangitis on the serum CA 19-9 levels; as was reported in literature, almost all of patients with hilar cholangiocarcinoma have hyperbilirubinemia, obstructive jaundice and cholangitis in turn arouses an enhancement in preoperative serum CA 19-9 levels (Harder et al. 2007; Rerknimitr et al. 2013; Juntermanns et al. 2010). Meanwhile, there is also little knowledge about the predictive value of serum tumor markers such as CA 19-9, CA 125 and CEA for evaluating tumor resectability in patients with hilar cholangiocarcinoma and the prognostic effect of various preoperative tumor factors on tumor resectability is also unclear. Thus, the role of serum CA 19-9, CA 125 and CEA in evaluating resectability of hilar cholangiocarcinoma should be further studied.

Therefore, the current study was conducted to highlight following points: (1) to examine the utility of preoperative CA 19-9, CA 125 and CEA levels in predicting the resectability of patients with hilar cholangiocarcinoma using the ROC analysis, (2) to study the correlation of various preoperative factors with tumor resectability rate using univariate and multivariate analysis.

## Patients and methods

### Patient selection

A total of 471 patients with radiographically potential resectable hilar cholangiocarcinoma at West China Hospital of Sichuan University from 1995 to 2014 were included. Patients with ampullary carcinomas, gallbladder carcinomas, intrahepatic cholangiocarcinomas were excluded, patients with radiographically unresectable neoplasm at presentation, preoperative chemotherapy and radiotherapy, preoperative serum CA 19-9 levels <5 U/ml (CA 19-9 nonsecretory), cholangitis were also excluded. For resectable tumors, pathological examination of the resected specimen was conducted, and for unresectable tumors, intraoperative biopsy was implemented. All patients were histologically identified as hilar bile duct adenocarcinoma.

### Pre-operative work-up

Before surgery, an elaborative medical history and physical exam was conducted in all patients, then the laboratory tests, the ultrasonography, the multi-detector row spiral CT, the magnetic resonance imaging were regularly detected. Preoperative biliary drainage was applied in patients with obstructive jaundice by endoscopic retrograde cholangiopancreatography (n = 142) and percutaneous transhepatic cholangiodrainage (n = 78). Patients were considered as unresectable if: (1) advanced bile duct infiltration that precludes intact tumor removal, (2) invasive of major vascular systems such as bilateral portal vein involvement that hampers vascular reconstruction, (3) lymph nodes metastases beyond the hepatoduodenal ligament, (4) unilateral hepatic lobe atrophy combined with invasion of contralateral portal vein or hepatic artery, (5) unilateral hepatic lobe atrophy combined with invasion of contralateral secondary biliary radicles, (6) unilateral secondary biliary radicles involvement combined with invasion of contralateral portal vein or hepatic artery, (7) pathology confirmed hilar cholangiocarcinoma with evidence of distant metastases (Dumitrascu et al. 2013). All the operations were conducted by experienced surgeons in our center.

### Data collection

The demographics, clinical data, laboratory data, operating details and survival outcomes were documented in detail. Serum CA 19-9, CA 125 and CEA levels were calculated by Roche chemiluminescence immunoassay method. Preoperative serum CA 19-9, CA 125, CEA levels were assayed just before the surgery so as to avert the influence of obstructive jaundice. Tumor size was determined as the largest dimension based on preoperative imaging test. Patients with negative margins (R0 resection) and microscopically positive margins (R1 resection) were defined as resectable, while those with macroscopically positive margins (R2 resection) and palliative surgery were considered as unresectable. Patients were then further sub-divided into two groups: patients with preoperative hyperbilirubinemia (n = 333) and patients with normal range of serum bilirubin levels (n = 138).

### Follow-up

As hilar cholangiocarcinoma is a devastating malignant disease, all patients were rigorously monitored and followed-up in outpatient clinics. All enrolled patients were assessed every 2–3 months with the basic measurement of tumor markers, liver functions, ultrasonography in the first year after surgery, and then 3–6 months annually thereafter. For those underwent curative surgery, if suspected as recurrence or distant metastasis, computed tomography or magnetic resonance imaging test was further conducted.

### Statistical analysis

Patient characteristics were expressed using frequency or descriptive analysis. The linear regression model was conducted to examine the relationship between CA 19-9, CA 125, CEA and the preoperative total bilirubin levels. The receiver operating characteristic (ROC) curve analysis was used both in the whole group (n = 471) and in the subgroup with normal range of serum total bilirubin levels (n = 138) to analyze the prognostic effect of CA 19-9, CA 125, CEA on tumor resectability and the best cut-off values of CA 19-9, CA 125 and CEA. Sensitivity, specificity, positive predictive values (PPV) and negative predictive values (NPV) were computed for each cut-off point of CA 19-9, CA 125 and CEA. Univariate and multivariate logistic regression models were also performed to check the independent factors correlated with resectability of these patients. The P value <0.05 was deemed as significant. The Statistical analysis was presented using the SPSS version 16.0 (SPSS Inc. Chicago, IL, USA).

## Results

### Demographic and clinical characteristics

Although 471 patients with hilar bile duct carcinoma appeared to have potentially resectable tumors in the light of the preoperative imaging examinations, only 309 of them (65.6 %) were operated on with curative surgery; which included hilar bile duct resection (45 cases), left hemihepatectomy (118 cases), right hemihepatectomy (86 cases), left trisegmentectomy (34 cases), right trisegmentectomy (13 cases), and mesohepatetctomy (13 cases). Caudate lobe was conventionally removed (251 cases), except for some earlier cases of type I papillary carcinoma. The other 162 patients were identified as unresectable due to tumors widely involving bilateral intrahepatic bile ducts or bilateral portal vein involvement or distant lymph nodes metastases or peritoneal metastases. The median CEA, CA 125, and CA 19-9 levels for all patients were 3.37 ng/ml, 22.5 and 199 U/ml, respectively. In those with normal range of serum bilirubin levels, the resectability rate was 71 % (n = 98; 11, hilar bile duct resection; 46, left hemihepatectomy; 30, right hemihepatectomy; 6, left trisegmentectomy; 3, right trisegmentectomy; 2, mesohepatetctomy), and the median CEA, CA 125, CA 19-9 levels for patients with normal bilirubin levels were 2.65 ng/ml, 22.0 and 155.8 U/ml, respectively; The clinical characteristics of the selected patients are lined out in Table 1.

**Table 1:** Patient characters

Variable	All patients (n = 471)	Normal bilirubin patients (n = 138)
Age^a^	60 [26–82]	60 [32–79]
Gender/male (%)	266 (56.5)	65 (47.1)
Pre-operative CA 19-9 level (U/ml)^a^	199 [5.97–3015.17]	155.8 [11.7–1000]
Pre-operative CA 125 level (U/ml)^a^	22.5 [1.23–456.2]	22.0 [2.53–171.4]
Pre-operative CEA level (ng/ml)^a^	3.37 [0.2–113.5]	2.65 [0.2–113.5]
Pre-operative TB level (μmol/l)^a^	167.5 [1.9–753.1]	27.5 [1.9–34.2]
Pre-operative ALT level (U/l)^a^	102 [8–772]	86.5 [10–720]
Pre-operative AST level (U/l)^a^	90 [11–1016]	83 [11–523]
Pre-operative Albumin level (g/l)^a^	37.2 [18.7–51.8]	38.2 [28.8–50.1]
Tumor size (cm)^a^	3 [0.8–15]	2.8 [0.8–6]
Preoperative biliary drainage (%)	220 (46.7)	20 (14.5)
Bismuth–Corlette classification (%)		
Type I and II	233 (49.5)	77 (55.3)
Type III and IV	238 (50.5)	61 (44.2)
T stage (AJCC) (%)		
T1 and T2	218 (46.3)	82 (59.4)
T3 and T4	253 (53.7)	56 (40.6)
Surgical procedures (%)		
Resected	309 (65.6)	98 (71.0)
Unresected	162 (34.4)	40 (29.0)
Postoperative complications (%)	137 (29.1)	32 (23.2)

*CA 19-9* carbohydrate antigenic determinant 19-9, *CA 125* carbohydrate antigen 125, *CEA* carcino embryonie antigen, *TB* total bilirubin, *ALT* alanine aminotransferase, *AST* aspartate transaminase, *AJCC* American Joint Committee On Cancer

^a^Parameters are presented as median and range

In all patients, a positive relevance was detected between preoperative serum CA 19-9 and total bilirubin level (r = 0.180, P = 0.012), but not in CEA (r = 0.103, P = 0.149) and CA 125 (r = 0.103, P = 0.149).

### Survival

**Fig. 1:**
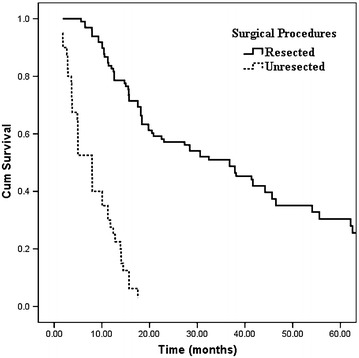
Kaplan–Meier curves comparing survival status based on surgical procedures (resectable and unresectable) in patients with potentially resectable tumors judged by radiologic examination (P < 0.001)

Then we further analyzed the survival outcome of patients with or without resectable tumors, The median follow-up time was 19 months (range 1–90 months). In resected group, the median overall survival time was 36.8 months and the 1, 3, 5 year survival rates were 83, 51, and 30 %, respectively, while in patients with unresectable diseases, the median overall survival time was 7.9 months and the 1, 3, 5 year survival rates were 28, 0, and 0 %, respectively (Fig. 1, P < 0.001).

### ROC analysis

**Fig. 2:**
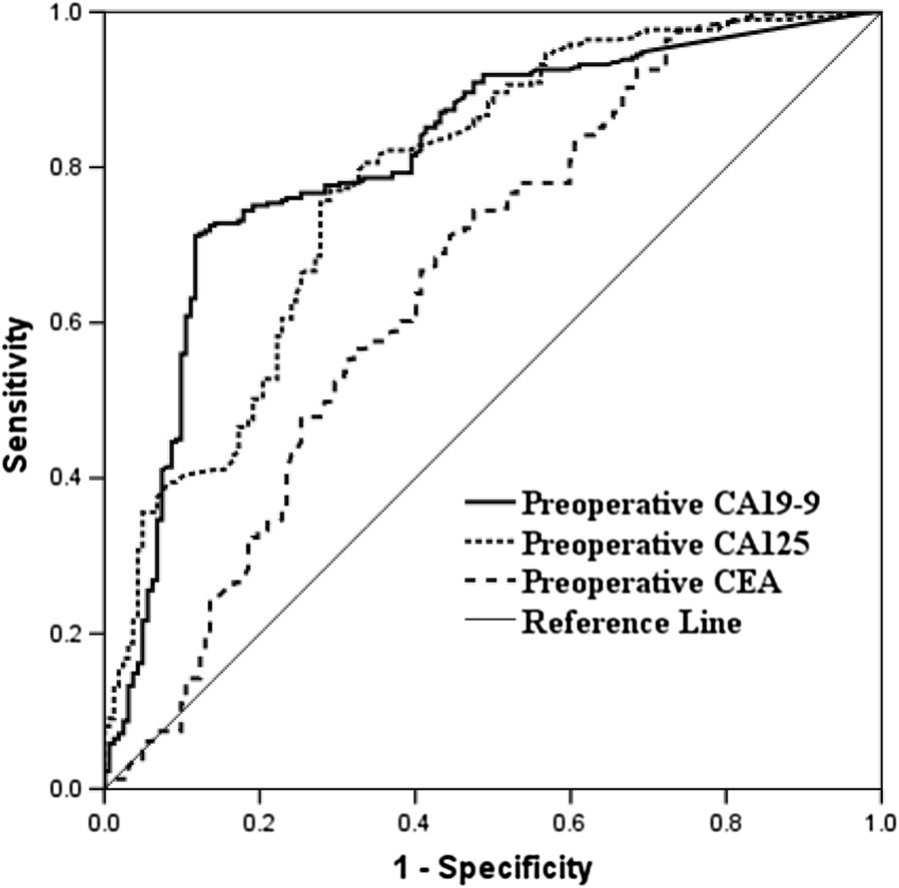
ROC analysis of CA 19-9, CA 125 and CEA for predicting the resectability of hilar cholangoicarcinoma in whole patients with potentially resectable tumors judged by radiologic examination

**Fig. 3:**
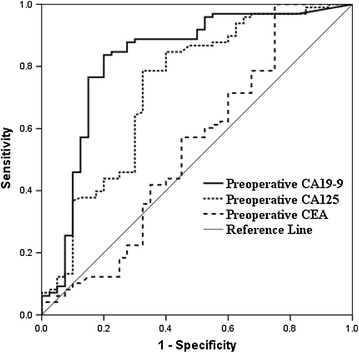
ROC analysis of CA 19-9, CA 125 and CEA for predicting the resectability of hilar cholangoicarcinoma in the subgroup of patients with normal range of serum bilirubin levels with potentially resectable tumors judged by radiologic examination

In the whole patients group (Table 2; Fig. 2), ROC analysis determined that the preoperative CA 19-9 cut-off value of 200.4 U/ml yielded a sensitivity of 71.2 %, specificity of 88.3 %, PPV of 92.1 %, NPV of 61.6 % in predicting resectability. In addition, a cut-off value of 26.84 U/ml for CA 125 also resulted in a sensitivity, specificity, PPV and NPV of 77, 71, 83.2 and 61.6 % for prediction of resectability respectively; Furthermore, in order to eliminate the impact of hyperbilirubinemia on CA 19-9 level, we then conducted ROC analysis in those with normal bilirubin levels (Table 2). We estimated the ideal CA 19-9 cut-off value of 203.96 U/ml in prediction of resectability, with a sensitivity of 83.7 %, specificity of 80 %, PPV of 91.1 % and NPV of 66.7 % (Fig. 3); meanwhile, the optimal cut-off value for CA 125 to predict resectability was 25.905 U/ml (sensitivity, 78.6 %; specificity, 67.5 %).

**Table 2:** ROC analysis of tumor markers to predict resectability of patients with potentially resectable hilar cholangiocarcinoma judged by radiologic examination

Parameter	For all patients (n = 471)			For normal bilirubin patients (n = 138)		
	CEA	CA 125	CA 19-9	CEA	CA 125	CA 19-9
Cut-off value	4.37	26.84	200.4	3.36	25.905	203.96
ROC area	0.659	0.785	0.815	0.542	0.730	0.825
95 % CI	0.604–0.713	0.740–0.829	0.773–0.857	0.425–0.659	0.629–0.830	0.736–0.913
Sensitivity (%)	74.4	77	71.2	60.2	78.6	83.7
Specificity (%)	52.5	71	88.3	45	67.5	80
PPV (%)	74.9	83.2	92.1	72.8	85.6	91.1
NPV (%)	51.8	61.6	61.6	31.6	56.2	66.7

*ROC* receiver operating characteristic, *PPV* positive predictive value, *NPV* negative predictive value, *CA 19-9* carbohydrate antigenic determinant 19-9, *CA 125* carbohydrate antigen 125, *CEA* carcino embryonie antigen

### Uni- and multivariate analysis the correlation of preoperative factors with resectability

The relationship of resectability rate with various preoperative tumor characteristics are presented in Table 3. In those with preoperative normal bilirubin levels, a significantly increased rate of preoperative CA 19-9 level >200 U/ml was observed in unresectable group compared with resected group (83.7 vs 16.3 %, P < 0.001). In addition, patients with preoperative CA 125 level >26 U/ml had a decreased resectability rate of 21.4 % in comparison with 78.6 % of those with lower CA 125 levels (P < 0.001). Meanwhile, the type 3 and 4 of Bismuth–Corlette classification also conferred to a poor resectability rate (37.8 %) when compared with type 1 and 2 (P = 0.017); Moreover, patients with BMI (body mass index) lower than the median value were also associated increased resectability (P = 0.03). Increased resectability was also witnessed in patients with preoperative tumor size ≤3 cm compared with tumor size >3 cm (P < 0.001).

**Table 3:** Analyzing the relationship of preoperative factors with the resectability rate in those with potentially resectable tumors judged by radiologic examination with normal bilirubin levels

Variable	Resectable (n = 98)	Unresectable (n = 40)	P value
Age^a^			
≤60	46 (46.9)	25 (62.5)	NS
>60	52 (53.1)	15 (37.5)	
Gender			
Female	47 (48)	26 (65)	NS
Male	51 (52)	14 (35)	
Symptom presentation			
No	38 (38.8)	18 (45)	NS
Yes	60 (61.2)	22 (55)	
Preoperative hospital stay >7 days^a^			
No	62 (63.3)	20 (52.6)	NS
Yes	36 (36.7)	18 (47.4)	
Preoperative CA 19-9 >200 U/ml^b^			
No	82 (83.7)	8 (20)	<0.001
Yes	16 (16.3)	32 (80)	
Preoperative CA 125 >26 U/ml^b^			
No	77 (78.6)	13 (32.5)	<0.001
Yes	21 (21.4)	27 (67.5)	
Preoperative CEA >3.4 ng/ml^b^			
No	59 (60.2)	22 (55)	NS
Yes	39 (39.8)	18 (45)	
Preoperative ALT level >50 U/l^c^			
No	25 (25.5)	16 (40)	NS
Yes	73 (74.5)	24 (60)	
Preoperative AST level >40 U/l^c^			
No	21 (21.4)	11 (27.5)	NS
Yes	77 (78.6)	29 (72.5)	
Preoperative albumin level >40 g/l^c^			
No	63 (64.3)	20 (50)	NS
Yes	35 (35.7)	20 (50)	
Tumor size^d^			
≤3 cm	76 (77.6)	18 (45)	<0.001
>3 cm	22 (22.4)	22 (55)	
BMI^a^			
≤21 kg/m^2^	66 (67.3)	19 (47.5)	0.03
>21 kg/m^2^	32 (32.7)	21 (52.5)	
Bismuth–Corlette classification			
I and II	61 (62.2)	16 (40)	0.017
III and IV	37 (37.8)	24 (60)	

*NS* not significant, *CA 19-9* carbohydrate antigenic determinant 19-9, *CA 125* carbohydrate antigen 125, *CEA* carcino embryonie antigen, *ALT* alanine aminotransferase, *AST* aspartate transaminase, *BIM* body mass index

^a^Using the median value

^b^Using the the best cut-off point in ROC analysis

^c^Using the lowing limit of the normal range

^d^Using the cut-off recommended in the DeOliveira staging system

To estimate the independent contributing determinants to the resectability, a multivariate logistic regression model was carried out and factors significant in the univariate analysis were all involved in (Table 4). It indicated that tumor size ≤3 cm (OR 4.149, 95 % CI 1.326–12.981, P = 0.015), preoperative CA 19-9 level ≤200 U/ml (OR 20.324, 95 % CI 6.509–63.467, P < 0.001), preoperative CA 125 levels ≤26 U/ml (OR 8.209, 95 % CI 2.624–25.677, P < 0.001) were independent determinants of resectability in patients diagnosed as hilar cholangiocarcinoma.

**Table 4:** Variables associated with tumor resectability in multivariate logistic analysis in those with potentially resectable tumors judged by radiologic examination with normal bilirubin levels

Variables	Odds ratio	95 % CI	P value
Tumor size >3 cm	4.149	1.326–12.981	0.015
Preoperative CA 19-9 >200 U/ml	20.324	6.509–63.467	<0.001
Preoperative CA 125 >26 U/ml	8.209	2.624–25.677	<0.001
BMI >21 kg/m^2^	2.131	0.696–6.528	0.185
Bismuth–Corlette (type 3 and 4)	1.857	0.629–5.487	0.263

*CI* confidence interval, *CA 19-9* carbohydrate antigenic determinant 19-9, *CA 125* carbohydrate antigen 125, *BMI* body mass index

## Discussion

Hilar cholangiocarcinoma is still a technically challenging operation characterized by low resectability rate, high postoperative recurrence, and poor survival outcome. Due to technologic progresses in preoperative imaging, much experience has been gained in preoperative assessment of the stage and aggressiveness of hilar bile duct tumors. However, many patients are still characterized as radiologically resectable tumors and undergo unnecessary laparotomy, only to be found to have advanced diseases such as tumors widely involving bilateral intrahepatic bile ducts or bilateral portal vein involvement or distant lymph nodes metastases or small peritoneal metastases that preclude curative resection. Hence, simple and practical methods like preoperative tumor marker evaluations are badly demanded to act as a complementary instrument to the preoperative imaging estimate for assessing the resectability of hilar cholangiocarcinoma so as to provide an additional reference and guideline for treatment of hilar cholangiocarcinoma.

CA 19-9, CEA, CA 125 are the most investigated tumor markers that have been appraised for preoperative diagnosis and postoperative prognosis in patients with hilar cholangiocarcinoma. Increased CA 19-9, CEA levels have been reported to be associated with advanced tumor stage and poor survival outcome (Juntermanns et al. 2010). Moreover, the two tumor markers were also widely used in the diagnosis of hilar cholangiocarcinoma (Rerknimitr et al. 2013; Soares et al. 2014; Nakeeb et al. 1996; Patel et al. 2000; Qin et al. 2004). Meanwhile, the dynamics of tumor markers are routinely detected as a measurement of recurrence and method to predict the disease process. Significantly elevated postoperative CA 19-9 level was associated with early recurrence (LaFemina and Jarnagin 2012). Nevertheless, little is known about the impact of these tumor markers on resectability of hilar cholangiocarcinoma.

In our current study, we demonstrated serum CA 19-9 was a predictor of resectability with an area under the curve (AUC) of 0.825, and the optimal cut-off value was 203.96 U/ml. Different from many previous studies, these results were not affected by hyperbilirubinemia and cholangitis, which was covered to have positive enhancement in CA 19-9 levels and was re-identified here—a positive relevance (r = 0.180, P = 0.012) was detected between preoperative serum CA 19-9 and total bilirubin level (Rerknimitr et al. 2013; Harder et al. 2007; Katz et al. 2010). In addition, patients with CA 19-9 nonsecretory were also excluded. Even in the total patients group (n = 471), which patients with hyperbilirubinemia were included, the preoperative CA 19-9 levels could also predict resectability with an AUC of 0.815. Furthermore, we conducted univariate and multivariate models to better estimate the predictive role of preoperative CA 19-9 with tumor resectability using the 200 U/ml cut-off point (to consistent with the ROC results). Patients with resected disease obviously had preoperative CA 19-9 lesser than 200 U/ml and CA 19-9 was also identified as an independent factor to be associated with tumor resectability. Thus, in consideration of the noteworthy predict value of CA 19-9 on resectability, it may act as a complementary tool to the preoperative imaging study so as to precisely predict the resectability of hilar cholangiocarcinoma and avoid unnecessary surgical attack.

Notably, enhanced preoperative CA 125 level was also correlated with decreased tumor resectability in hilar bile duct carcinoma in current series, as CA 125 is noted to be a momentous factor in detection and management of ovarian cancer (Chen et al. 2015). Besides, CA 125 also has prognostic effect on pancreatic cancer and can also predict the resectability (Chen et al. 2014; Luo et al. 2013; Gu et al. 2015). However, no previous studies have specifically discussed the predictive value of CA 125 on resectability of hilar cholangiocarcinoma. In the present study, CA 125 was not associated with hyperbilirubinemia and cholangitis. In the whole patients group, an AUC of 0.785 was identified and a best cut-off value of 26.84 U/ml was also obtained. Even in patients with normal bilirubin level, the CA 125 remained predictive of resectability with an AUC of 0.730 and a optimal cut-off value of 25.9 U/ml. The predictive value of CA 125 was definitely lower than the CA 19-9. Using the cut-off point of 26 U/mL, patients with CA 125 levels larger than 26 U/ml were significantly correlated with lower resectability as was confirmed in the multivariate regression model in current study. Thus, together with CA 19-9, the CA 125 should be preoperatively analyzed and regarded as a supplementary kit to make up for the defection of preoperative imaging test in predicting the resectability and better guide the treatment.

The relationship of CEA and resectability was also analyzed. In patients with normal bilirubin levels, ROC analysis determined that the preoperative CEA cut-off value of 4.37 ng/ml yielded a sensitivity of 60.2 % and specificity of 45 % in predicting the resectability. In the univariate and multivariate logistic analysis, the CEA level was not a reliable factor associated with resectability. Thus, the predictive value of CEA on tumor resectability was less than CA 19-9 and CA 125.

In addition, tumor size was also carefully examined and it was associated with tumor resectability. Patients with tumor size >3 cm were more likely to possess an unresectable tumor. It has been universally acknowledged that patients with resectable disease undoubtedly have preferable survival outcome and was re-confirmed in this series (Dumitrascu et al. 2013; Dinant et al. 2006; Lee et al. 2010). In present study, we used the tumor size collected from preoperative imaging findings, as post-operative pathological tumor size finding was difficult to evaluate in cases of unresectable tumors and it depends on the experience of the surgeons, which may induce a bias. Thus, in view that the 3 cm cut-off point could affect the resectability, we may conclude that tumor size was also an important prognostic factor. On the other hand, the 3 cm cut-off also identified the usefulness of the current T stage of DeOliveira staging system, in which the 3 cm cut-off was characterized as T3 (Deoliveira et al. 2011; Regimbeau et al. 2014), patients with T3 tumors are not eligible for liver transplantation and have a high tendency of accompanying with poor outcome (Chaiteerakij et al. 2014; Hassoun et al. 2002; Darwish Murad et al. 2012). In addition, the T3 tumors frequently invade the major vascular tissues, the caudate lobe, and the peripheral nerve system and make the resection extraordinary difficult and more often than not, patients in this situation have unresectable disease as was confirmed here. Thus, the preoperative tumor size should also be taken into account to better judge the resectability.

Under most circumstances, it is unpractical to employ a single biomarker to forecast the resectability of hilar cholangiocarcinoma. Tumor biomarkers should act as complementary instrument to the preoperative imaging evaluation, and a comprehensive combination of clinical manifestation, preoperative imaging scan and other prognostic factors including the tumor size are urgently warranted so as to better assess the resectability of hilar cholangiocarcinoma and provide a pivotal reference and guideline for treatment of hilar cholangiocarcinoma.

Our study has limitations inherent to its retrospective study design. Moreover, hilar cholangiocarcinoma is generally diagnosed at an advanced stage and many patients are with hyperbilirubinemia. Thus, patients with normal bilirubin levels are relatively less. Therefore, further research and multi-center studies are clearly warranted to support clinically usefulness of the present finding.

In conclusion, Preoperative CA 19-9 and CA 125 levels predict resectability in patients with radiological resectable hilar cholangiocarcinoma. Increasing preoperative CA 19-9 levels and CA 125 levels are associated with poor resectability rate, which may act as auxiliary indexes for assessing the resectability of hilar cholangiocarcinoma.

## Abbreviations

ROC: receiver operating characteristic analysis
CA 19-9: carbohydrate antigenic determinant 19-9
CA 125: carbohydrate antigen 125
CEA: carcino embryonie antigen
PPV: positive predictive value
NPV: negative predictive value
OR: odds ratio
AUC: areas under the curve
